# “It's not healthy and it's decidedly not masculine”: a media analysis of UK newspaper representations of eating disorders in males

**DOI:** 10.1136/bmjopen-2014-007468

**Published:** 2015-05-29

**Authors:** Alice MacLean, Helen Sweeting, Laura Walker, Chris Patterson, Ulla Räisänen, Kate Hunt

**Affiliations:** 1 MRC/CSO Social and Public Health Sciences Unit, University of Glasgow, Glasgow, UK; 2 HERG Health Experiences Research Group, Department of Primary Care Health Sciences, University of Oxford, Oxford, UK

**Keywords:** SOCIAL MEDICINE, MENTAL HEALTH

## Abstract

**Objectives:**

Recent qualitative research found young men reporting that an expectation that eating disorders (EDs) mainly affect young women led them, and others, to only recognise their symptoms when their ED had become entrenched. This raises questions about how these stereotypes persist. We therefore explored how EDs in males were represented in articles published in UK newspapers over a 10-year period (7.12.2002–7.12.2012), specifically attending to whether newsprint media represent EDs in males as ‘gender appropriate’, ‘gender anomalous’ or ‘gender neutral’.

**Design:**

A qualitative thematic analysis of UK newspaper articles.

**Methods:**

We searched two databases, Newsbank and LexisNexis, for newspaper articles including ED and male terms in the lead/first paragraph. Following de-duplication, 420 articles were scrutinised; 138 met inclusion criteria for detailed textual analysis and were imported into NVivo10.

**Findings:**

The number of articles peaked in 2008 when a UK politician announced that he had experienced bulimia nervosa. Analysis of how the articles portrayed male ED-related characterisations and experiences revealed that they conveyed ambiguous messages about EDs in males. Despite apparently aiming to dispel stereotypes that only young women experience EDs and to address stigma surrounding EDs in males, many aspects of the articles, including repetition of phrases such as ‘a young woman's illness’, serve to reinforce messages that EDs are inherently ‘female’ and so ‘anomalous’ for men.

**Conclusions:**

Newspaper articles represent men with EDs as *atypical of men*, as a result of having an ED (and any feminising or demasculinising characteristics associated with this), *and* as *atypical of people with EDs*, who are still usually portrayed as teenage girls. Such media representations frame a cultural paradigm in which there is an expectation that men may feel shame about or strive to conceal EDs, potentially contributing to men with EDs delaying help-seeking, gaining late access to treatments and reducing chances of successful outcomes.

**Strengths and limitations of this study**

- This is the first study to explore in detail how newspaper articles represent eating disorders in males.
- It examined coverage from 10 UK newspapers over a 10-year period.
- It did not examine other popular media sources (eg, magazines, websites, television) which may cover different topics and/or be more widely publicly accessed.
- It did not examine audience reception, although the analysis was informed by recent qualitative research suggesting that men may delay help-seeking for symptoms because of low recognition of eating disorders in men.

## Introduction

Eating disorders (EDs) have been highlighted as a clinical priority area for Youth Mental Health in 2013–2016.^1^ They can prove fatal if not treated early, and anorexia nervosa (AN) has the highest mortality rate of all psychiatric conditions in adolescence.^2^ Although its prevalence is higher in women, the literature suggests that men constitute around 25% of community-based samples meeting ‘full’ ED criteria, but 10% or less within diagnosed samples. 3–5 In the UK, an English adult general population survey found 9.2% of women and 3.5% of men screened positive for EDs, based on a threshold justifying clinical assessment.^6^ Analyses of UK (2009) primary care data among 10–49-year-olds showed that incidences for all EDs per 100 000 population were: 62.7 for women and 7.1 for men.^7^ Studies of specialised UK ED services have reported that 11%^8^ and 5%^9^ of patients were men.

The smaller proportion of males generally identified in clinic-based compared with community-based samples suggests a “*community reservoir of undiagnosed men*” (ref. 10, p.20). EDs in males have been said to be “*underdiagnosed, undertreated and misunderstood*”^3^ and there is a paucity of research on men's ED-related experiences.^11^ We recently reported on a UK-based qualitative study of young people with an ED, and in particular on the experiences of young men.^2^ These men reported confronting perceptions of EDs as uniquely, or very largely, ‘female illnesses’, which contributed to them being slower to recognise their own patterns of behaviours as ED symptoms. Consequently, many presented late in their illness trajectory when behaviours and symptoms had become entrenched, and hence more difficult to treat. Some felt that family, health professionals and others (eg, teachers) had also missed opportunities to recognise their illness because of a widespread cultural construction of EDs as a ‘female illness’.^12–15^ Although some illnesses, such as testicular or ovarian cancer, only affect one sex, there are others which can affect both, but are more, and sometimes much more, common in one (eg, breast cancer, autism, multiple sclerosis to varying degrees). In the context of such disparities in prevalence, an illness could theoretically be portrayed as ‘gender appropriate’ (eg, breast cancer in women), ‘gender anomalous’ (eg, breast cancer in men^16^ ^17^) or as ‘gender neutral’ (eg, multiple sclerosis). This can affect how symptoms are experienced, recognised and treated.^2^ ^18^

Given evidence that men constitute around a quarter of those with EDs, the question is how and why EDs are still seen as almost exclusively ‘female illnesses’. One contributory factor may be the mass media, an important source of cultural references and health information.^19–22^ The way news items are filtered and constructed can impact the understanding of the issues being reported. Story selection and prominence may influence readers’ views of the importance of particular issues, while the way a story is framed may affect how audiences make sense of issues. These frames often reflect broader cultural themes^23^ ^24^ and can be powerful when presented consistently over time.^25^

Despite declining circulations, newspapers (and their online versions) remain an important source of public information, although the volume and quality of reporting of health-related research varies widely in UK newspapers.^26^ We are aware of three studies of EDs in newspapers. However, apart from noting a lack of focus on men, none address newspaper representations of EDs in males.^13^ ^27^ ^28^

Against this background, we examine representations of men with EDs in newspaper articles over a 10-year-period. In the context of widespread reporting of a higher prevalence of EDs in (young) women,^5^ our aim is to investigate whether EDs in men are presented in ‘gender neutral’ terms, or as either ‘*gender appropriate’* or ‘*gender anomalous’* for men.

## Methods

### Newspaper selection

Ten national UK newspapers, with a range of readership profiles, including multiplatform circulation and readership, were selected for this study.^29^ ^30^ We included: three ‘serious’ (‘broadsheet’) newspapers including their Sunday and online counterparts *(The Guardian [TG], Guardian Unlimited [GU]* and *The Observer [TO]; The Independent [TI], The Independent on Sunday [IOS]* and *independent.co.uk [IO]; The Daily Telegraph [DT], The Sunday Telegraph [ST]* and *telegraph.co.uk [DTO]);* two ‘middle-market tabloid’ newspapers (the *Daily Mail [DMa],* the *Mail on Sunday[MOS]* and the *Mail Online [MO]; The Express [TE]* and *The Sunday Express [SE]);* and five ‘tabloid’ newspapers *(The Sun [TS]; The News of the World [NW—ceased publication July 2011];* the *Daily Star [DS]; The People [TP];* the *Daily Mirror [DMi]* and the *Sunday Mirror [SMi]).* This typology has been used in similar analyses^31^ ^32^ to identify a broad newspaper sample with various readership demographics and political orientations, thus capturing the potential range of ways that print media frame particular topics.

### Search strategy

We selected a 10-year-period, from 7.12.2002 to 7.12.2012, because this represented a time of increasing academic and clinical interest in males with EDs^11^ and we anticipated would yield a substantial number of articles for analysis. Articles in the target publications were identified using the electronic databases *LexisNexis* and *Newsbank* using search terms relating to: EDs (“eating disorder”, “eating issue”, “eating problem”, “anorex*”, “bulim*”, “manorex*”, “bigorex*”, “orthorex*”, “binge eat*”, “compulsive over eat*”, “eating disorder not otherwise specified”, “EDNOS”, “over eating disorder”, “disordered eat*”, “b-eat”^i^) and males (“male”, “m*n”, “boy”, “lad”, “bloke”, “guy”). Wild cards were included to capture variants such as anorexic/anorexia, eat/eater/eating and man/men. To maximise the likelihood of identifying articles which were substantially focused on EDs in males, we identified search terms within text at the beginning of articles (specifying ‘at the start’ in *LexisNexis* and ‘lead/first paragraph’ in *Newsbank*). Note that early searches using fewer ED terms (“eating disorder”, “eating issue”, “eating problem”, “anorex*”, “bulim*”) without male terms or limiting the search to the beginning of articles identified 16 232 articles. We therefore included these restrictions in future searches.

The searches (conducted by LW) identified 480 articles; 420 remained following de-duplication. Each article was read closely by LW who initially worked with HS in respect of 40 articles to establish agreement over relevance of content to the research question; by the end of this process, agreement in relation to the content of these articles was almost perfect. Decisions on inclusion/exclusion of a further 26 articles where there was any uncertainty about eligibility were made in discussion between LW and HS. Articles were excluded if they: (1) contained no text acknowledging that males can suffer from EDs: (2) only used an ED term as an adjective or metaphor (eg, ‘slim to anorexic chance of winning’); (3) profiled a male celebrity who had an ED, but in which this was only tangentially mentioned; (4) were short lead-ins referring to a main article in the same newspaper edition (main article hence included in the sample); or (5) were letters, problem pages/advice, TV guides or review pages. On these criteria, 138 articles were identified for detailed textual analysis.

### Data extraction and analysis

All 138 included articles were read by multiple authors to generate five broad thematic categories (male ED-related characterisations: experiences; prevalence; aetiology; explicit gender comparisons). This broad coding schema was applied (by LW) using NVivo10 software so that all text which pertained to these five themes could be analysed in close detail (see below). In practice, this broad coding (eg, around 50% of the total text in the 138 articles was coded as ‘experiences’ and around 30% as ‘characterisations’) resulted in an extensive overlap: around 90% of ‘characterisations’ were also coded as ‘experiences’, while around 50% of experiences were also coded as ‘characterisations’. The ‘gender comparisons’ theme proved redundant once the ‘experiences’, ‘characterisations’ and ‘prevalence’ (reported elsewhere^5^) themes had been analysed in detail. Broadly, ‘characterisations’ focused on ages, sexuality and personal features of men with EDs and ‘experiences’ on manifestations, treatment, legacy and the perspectives/voices represented in the articles.

For our detailed interpretive analysis of the experiences and characterisation themes, four authors (AM, HS, UR, KH) analysed and summarised all material coded to the themes. Initially, two authors worked independently on each theme, using the ‘One Sheet Of Paper’ (OSOP) method.^33^ This involves a close reading of all data coded to a theme, noting, under separate headings, all instances of subthemes or issues raised and identifying each note or quote (in this case via newspaper article reference numbers). This ensures a systematic approach, including noting anticipated (eg, in this analysis, whether EDs in men were portrayed as gender ‘neutral’, ‘appropriate’ or ‘anomalous’) and unanticipated themes, and enables attention to be paid to ‘deviant cases’ to ensure that all perspectives are captured.^34^ The researcher pairs then discussed their independent interpretations to produce a mutually agreed definitive descriptive summary for each theme. All authors then examined how the themes inter-related; this revealed an underlying tension in the data between framing of EDs in men as ‘gender appropriate’ or ‘neutral’ (emphasising that men *can get* EDs) and those which forefronted a ‘gender anomalous’ framing.

## Findings

Following a description of the articles, our findings are structured in terms of messages that males *can get* EDs and then those which appeared to reinforce underlying messages that *EDs are ‘gender anomalous’* for men (EDs normally affect females; ED risk is greater among less masculine men; men find EDs shameful; EDs in men are not recognised by professionals). We end our findings with a ‘case study’ describing the reporting in relation to one prominent UK politician who revealed an ED, which highlights the portrayal of EDs as ‘*gender anomalous*’ for men.

### Description of included articles

Of the 138 articles meeting our inclusion criteria, 46 were published in ‘serious’ papers, 32 in ‘middle-market tabloids’ and 60 in ‘tabloids’ (table 1). Over half appeared in just four publications: one ‘serious’ (Guardian, 10.9%), one ‘middle-market’ (Daily Mail, 10.9%) and two ‘tabloid’ (Daily Mirror, 19.6%; The Sun, 12.3%) newspapers. Similar numbers appeared in the ‘news’ (51%, average words=398) and other (49%, average words=1639) sections of papers; as would be expected, those appearing in non-news sections (such as ‘features’) were longer.

**Table 1:** Articles (n=138) by newspaper genre and publication

Genre	Title of publication	N articles (%)
Serious	Guardian	15 (10.9)
	Observer	11 (8.0)
	Independent	5 (3.6)
	Independent on Sunday	1 (0.7)
	Independent.co.uk	2 (1.4)
	Daily Telegraph	8 (5.8)
	Sunday Telegraph	2 (1.4)
	Telegraph.co.uk	2 (1.4)
		**46 (33.3)**
Middle-market tabloid	Daily Mail	15 (10.9)
	Mail on Sunday	4 (2.9)
	Mail Online	6 (4.3)
	Express	4 (2.9)
	Sunday Express	3 (2.2)
		**32 (23.2)**
Tabloid	Daily Star	8 (5.8)
	Daily Mirror	27 (19.6)
	Sunday Mirror	2 (1.4)
	The People	4 (2.9)
	News of the World	2 (1.4)
	The Sun	17 (12.3)
		**60 (43.5)**
**Total**		**138 (100.0)**

**Figure 1:**
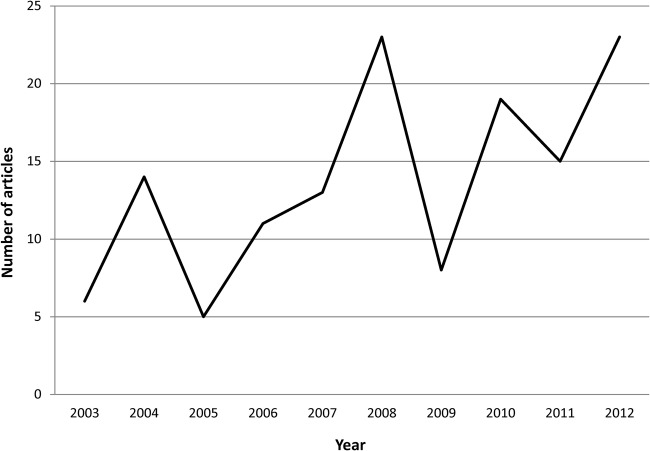
Number of articles by year (Dec 2002–Dec 2012).

These was some suggestion (figure 1) of an upward trend in the number of articles reported each year, from 6 in 2003 to 23 in 2012. A peak occurred in 2008 when half (n=12) the articles in that year reported on the revelation by John Prescott, UK politician and ex-deputy prime minister, then aged around 70 years, that he had experienced BN in middle age.

Most articles feature first-hand retrospective accounts from men with EDs, mainly recounting symptom onset and manifestation, including descriptions of both emotions and behaviours. Several refer to public figures and UK television personalities, who have spoken publicly about having experienced EDs. Relatively few feature first-hand accounts from men currently experiencing acute phases of EDs. Nearly half feature quotes from ‘experts’, such as charity spokespeople, nutritional consultants, psychiatrists and psychologists, describing emotions and behaviours which often characterise men's experiences of EDs. A small number also feature second-hand (mostly mothers’) accounts of boys’ and men's experiences of EDs.

### Males can get EDs

An apparently clear message within most articles was the need for increased awareness that males *can* experience EDs, exemplified by a quote from a 28-year-old amateur boxer “*addicted*” to dieting who wanted “*to be a warning to young boys out there. Some people might laugh at the thought of a guy with an eating disorder but it can easily take hold*” (NW-05/12/2010). Many articles suggested rising rates of EDs and/or body-image concerns among men, as they become “*more weight obsessed*” (TO-03/08/08), acquire “*body hang-ups*” (DMi-23/09/08) or replicate the “‘*size zero’ trend*” previously seen in females (TO-16/05/10).

Articles tended to suggest that if men do develop an ED, this will more commonly happen during teenage and early adulthood years: “*male eating disorders are most likely to begin between the ages of 14 and 25*” (DMa 25/02/2010). Related to this, most articles including men's perspectives and experiences detailed the (young) age at which they believed their ED began; for example, “*As a child I never had any issues with food. But from my early teens I was plagued with feelings of negativity and worthlessness*” (DMa-14/11/2006). Relatively few articles suggested that EDs can be experienced by men at any age, although a few featured profiles of middle-aged men who had lived with EDs for many years; for example, one quoted a 55-year-old for whom “*anorexia nervosa has been a 30-year struggle*” (SE-07/06/2009).

However, having highlighted that men *can* suffer EDs, articles often subtly contradicted this message in several ways elsewhere. We describe these below (EDs normally affect females; ED risk is greater among ‘less masculine’ men; men find EDs ‘shameful’; EDs in men are not recognised by professionals) and end with a case study of the reporting of EDs in men.

### EDs normally affect females (so are ‘gender anomalous’ for men)

Rather than simply conveying that EDs remain more common in females, the language used generally reinforced normative cultural assumptions that EDs affect them (almost) exclusively, using phrases such as “*normally associated with girls*” (DMa-29/08/2003), “*seen as a young woman's illness*” (DMi-17/08/2004) and “*mainly affects teenage girls and young women aged 16 to 25*” (TI-22/04/2008). This repeatedly underlined the idea that EDs are not only stereotypically ‘female illnesses’, but one particularly affecting teenage girls and young women. The overall implication is that while EDs *can* occur in men and boys, they are nonetheless ‘gender anomalous’ for males.

### ED risk is greater among less masculine men (so in men who are not ‘gender typical’)

The second way that articles undermined the more general message that men can be affected by EDs was by implying that only *certain types* of men are at risk. The descriptions of men with EDs often emphasised characteristics culturally stereotyped as antithetical to dominant forms of masculinity. For example, men with EDs were described as: feeling “*physically inadequate*” (DMi-03/08/2011); insecure about body shape and wanting “*more of a manly physique*” DMa-01/04/2006); or “*vulnerable young men*” (DMi-25/02/2010). One spokesperson is quoted as saying “*underneath, it's about being sensitive, having no perspective of when enough is enough, having low self-worth and being a perfectionist*” (DMa-2/06/07). The sense that men with EDs are weaker and have somehow failed to achieve what might be expected of them as men was often suggested in quotes from men themselves: “*No one expects a man, especially a successful one, to have an eating disorder. It seems such a weakness*” (DT-12/08/2009, quoting television personality Uri Geller); “*Young men who starve themselves look effeminate. It's not healthy and it's decidedly not masculine*” (DS-03/11/2006, quoting the chair of a parenting charity).

Related to this, again implying that only certain more marginalised groups of men are at risk, some articles discussed sexual orientation in relation to EDs, noting, for example, that “*gay men [are] more likely to be afflicted*” (SE-28/03/2004), “*a belief that male sufferers are predominantly gay*” DMi-23/03/2011) or the “*suggestion that they [men with EDs] will be gay or overly feminine*” (DMi-23/09/2009). Even articles featuring accounts from heterosexual men with EDs called their heterosexual credentials into question through inclusion of observations about being ‘unlucky’ in love, unable to attract or “*rubbish”* with women (TG-27/02/2007), with one describing those seeking help as “*coming out about”* their ED (DMi-23/03/2011). A spotlight on some heterosexual sportsmen's experiences of EDs is interesting, as culturally these men would typically be ascribed high status in archetypical hierarchies of masculinity. However, the articles suggested that performance of masculinity is precarious even for these men if they are known to have an ED: for example, “*The revelation [that racing driver David Coulthard experienced BN] is a surprise on two counts. First, Coulthard is a sportsman, albeit in a discipline that requires a certain sleekness […] Perhaps even more surprising is Coulthard's maleness […] bulimia has until recently been considered a largely female disease*” (TG-22/08/2007). Describing David Coulthard's BN as “*a surprise*”, because he is male *and* an elite sportsman, again reinforces the notion that EDs are ‘gender anomalous’ for males.

### Men find EDs shameful (because they are ‘gender anomalous’)

A third way in which articles conveyed a sense that EDs are ‘gender anomalous’ for males was by reinforcing the message that men with ED symptoms who require professional care are ‘weird’ as men. Around half the articles detailing men's experiences commented on how difficult, or even shameful, it is for them to seek help for EDs. Men were repeatedly described as “*ashamed*” (DMa-11/04/2012), “*very slow to admit they have a problem*” (DMi-17/08/2004) and “*liv[ing] in fear of stigma*” (IO-11/10/2012). Articles featuring first-hand accounts often explicitly linked these feelings to masculine identities or to the construction of EDs as a ‘female illness’: “*many men are too ashamed to admit they suffer from a disease usually associated with women […] having anorexia as a boy makes you seem weak*” (DMi-25/04/2012); “*admitting to eating disorders isn't macho*” (DMa-21/07/2011). The use of phrases such as “*own up*” (DMa-23/12/2008), “*confess*” (DMi-01/12/2009) and “*admitting*” (DMi-17/08/2004) suggests men who seek help for EDs risk being seen as doing wrong or transgressing masculine norms.

### EDs in men are not recognised by professionals (because they are ‘gender anomalous’)

Finally, the relatively small number of articles referring to experiences of seeking medical help may also have undermined the message that men *can be* affected by EDs, by portraying GPs as dismissive of, and/or reluctant to diagnose men with EDs. These articles suggest that this is because health professionals also associate EDs with women. For example, one spokesperson is quoted as saying: “*Doctors, just like anyone else, tend to see eating disorders as a woman's illness*” (DMi-25/02/2010). A number of articles also highlight the ways in which, when men do seek information or help, they have different and more negative experiences than women. For example, one quotes a 19-year-old male with AN as saying, “*all the literature and self-help guides on the subject, their weight tables and lists of symptoms are written for women […] this only enhanced my feelings of isolation and shame […] society seemed to view my illness as inherently feminine*” (TG-26/02/2007). Few focused on men's experience of ED treatment, and only one of these included positive details about his therapy: “*Thanks to skilled counselling and finding the right kind of help, I have finally been able to beat anorexia*” (DMa-14/11/2006).

### A case study of the reporting of EDs in men

In summary, many articles which ostensibly purported to be raising awareness that EDs can occur in men of any age often conveyed at best a mixed message, emphasising a cultural assumption that men are, and *should be*, ashamed to admit they have a (young) woman's illness. Many of the articles reporting John Prescott's experiences demonstrate this well; the language used is ambiguous and reinforces the construction of EDs as ‘gender anomalous’ for men and the opprobrium that men, especially older men, can expect if they ‘admit’ to ED symptoms. One article, headlined “*John Prescott is the Princess Di of politics*” (Princess Diana also spoke publicly about her bulimia), describes how “*The girl from a stately home and the boy who failed his eleven-plus both grew into adults who rushed from official functions to throw up*” (TI-28/05/2008). Nearly all the 21 articles reporting on Prescott's experiences explicitly note his age. While a few use his experience to show “*anyone can develop an eating disorder at any age*” (DMa-21/04/2008), most express great surprise, including from expert spokespeople, that an ED could be experienced by a man of Prescott's age: “*I've never come across a man this old with bulimia*” (DMi-21/04/2008). The articles express concern that “*there could be thousands of middle-aged men secretly struggling with a terrible condition*” (DMi-21/04/2008) and speculate that “*maybe we're missing a whole audience of middle-aged men who are too scared to admit they have a problem*” (DMa-21/04/2008). Even articles which apparently praise Prescott's ‘courage’ in revealing his experiences imply that men who do not wish to have their masculinity publicly policed or called into question should not reveal that they have this ‘female illness’:

John Prescott wins our praise for his bravery in admitting his battle with bulimia. It cannot be easy for a proud man who values his political reputation to admit to an eating disorder. […] He talked openly and honestly about a disorder which many believe only affected young women and teenage girls. […] No doubt the mockers will poke fun and his friends are worried that the admission will damage the way he is seen by history. (DMi-21/04/2008).

## Discussion

Academic and clinical interest in males with EDs has increased since the 1990s, with growing numbers of research papers and specialised ED services.^11^ Rising numbers of newsprint articles on EDs in males between 2002 and 2012 reflect this. Our analysis of these articles highlighted several key messages about how boys and men with EDs are described and characterised in the public domain. One key message is their lack of visibility: over a 10-year period, only 138 articles were identified as relevant to our research question of whether newsprint media represent EDs in males as ‘gender appropriate’, ‘gender anomalous’ or ‘gender neutral’. Most articles, we would argue, communicated (perhaps unwittingly) competing messages, raising awareness that males can get EDs while reinforcing underlying messages that EDs are a ‘woman's’ illness. This assumption, that ‘real men’ should not experience EDs, was one which recent qualitative research showed young men with EDs were highly conscious of.^2^ Articles often suggested that, when EDs *do* occur in males, only adolescent or younger men are affected and that men with EDs are effeminate or less manly, and are (or ‘should’ be) ashamed to seek help. Some first-hand accounts also included negative experiences of help-seeking and treatment, or of feeling disbelieved or dismissed. Thus, many key messages within the articles were riven with ambiguity. On the one hand, they suggested EDs are ‘possible’ and indeed increasingly common in young men and boys; on the other, they were portrayed as sufficiently rare among middle-aged or older men to be ‘weird’ enough to be almost inconceivable. Despite, on one level, ostensibly aiming to dispel the stereotype that only young females experience EDs, many aspects of the articles served to *reinforce* messages that EDs are inherently ‘female’. Males with EDs were thus portrayed as ‘anomalous’ in two ways; as *atypical of men* and as *atypical of those with EDs*, with the teenage girl still being portrayed as not only the typical ED sufferer in a statistical sense, but also from a normative perspective.

Newspaper representations have therefore tended to frame a cultural paradigm in which there is an expectation that men may feel discomfort, even shame, about having an ED and so may strive to conceal it. This may in turn contribute to men with EDs delaying help-seeking, gaining late access to treatments and hence reduced chances of successful outcomes, even when they do recognise their symptoms, as described in recent qualitative research on young men with EDs.^2^ At the very least, strong cultural constructions of EDs as feminised may make the negotiation of pathways to care more complicated for men with EDs^9^ ^35^ and may reduce the likelihood of them seeking help and treatment.^36^ A delay in men's help-seeking can be compounded by a lack of awareness of EDs in men among primary healthcare practitioners,^37^ reinforcing other evidence that suggests that patient gender affects the interpretation of the meaning of signs and symptoms presented in general practice.^38^ ^39^

This study is the first to explore in detail how EDs in males are represented in newspaper articles, but has some limitations. Although our analysis examined a 10-year-period and we were able to explore coverage from 10 UK newspapers, we were not able to examine other media sources, such as magazines, websites or television, which may cover different topics and/or be more widely accessed by the public. Nor was the study designed to examine audience reception, so it does not explore how the messages were interpreted by different audiences. However, our recent analysis of the experience of young men with EDs reflects remarkably similar views about how men with EDs are seen in contemporary Britain.

The ways in which news media affect lay understandings of illness are thought to be “*complex and diverse*” (ref. 40, p.569), yet media representations should be critically examined because of their potentially powerful influence on individuals’ attitudes, beliefs and perceptions about health issues,^20–22^ and perceptions of being ‘at risk’ or a ‘candidate’ for particular illnesses. Social constructions of illness have an important impact on the ways in which symptoms of illness are experienced, recognised and presented to health professionals, as well as interpreted and treated by them. Media representations of EDs are likely to contribute in important ways to the culturally available ‘illness representations’ and ‘stocks of knowledge’ which people draw on to make sense of their symptoms and decide on appropriate actions.^41–44^ Thus, exploring media representations of males with EDs is important in order to understand how they contribute to the cultural construction of EDs as ‘gender appropriate’ for females but ‘gender anomalous’ for males.

We conclude that while newsprint representations highlighting EDs in males may serve to increase awareness and so lessen associated stigma, the ways these ideas are conveyed may have significant implications for the experiences, help-seeking and treatment of men with EDs. It could be argued that those responsible for media portrayals of EDs in males are faced with negotiating a difficult balance, as current research evidence suggests that women are more likely to suffer from EDs,^3^ ^4^ that homosexuality, bisexuality and gender-identity confusion are risk factors for EDs in males^8^ ^36^ and possibly that EDs are more prevalent among men who report traits more typically associated with femininity, such as unassertiveness and low self-esteem, than among more ‘masculine’ and ‘androgynous’ men.^45–47^ Our analysis points to several potential recommendations for media reporting of this topic, including a more neutral commentary on the prevalence, careful scrutiny of terms and removal of those which emphasise EDs as ‘gender anomalous’ for males (eg, ‘young women's illness’, ‘vulnerable’, ‘sensitive’, ‘overly feminine’, ‘ashamed’) and balanced reporting of treatment experiences. B-eat, the national UK EDs charity, has produced media guidelines for reporting EDs^ii^ which note the power of media influences and highlight the need for sensitive reporting. The B-eat website notes “*a welcome increase in accurate reporting in recent years*” and there is evidence that it is increasingly being used as a ‘go to’ source on EDs by UK newspapers.^5^ Our analysis suggests that a section focusing on (reporting of) EDs in males might be a useful addition to any such media reporting guidelines.

## Footnotes

i b-eat is a national UK eating disorders charity.

ii http://www.b-eat.co.uk/assets/000/000/072/BeatMediaGuidelines_original.pdf (accessed 12/03/2015).
